# Protocol of a randomised controlled, open-label trial of ex vivo normothermic perfusion versus static cold storage in donation after circulatory death renal transplantation

**DOI:** 10.1136/bmjopen-2016-012237

**Published:** 2017-01-23

**Authors:** Sarah A Hosgood, Kourosh Saeb-Parsy, Colin Wilson, Christopher Callaghan, Dave Collett, Michael L Nicholson

**Affiliations:** 1 Department of Surgery, University of Cambridge, Addenbrooke's Hospital, Cambridge, UK; 2 Freeman Hospital, Newcastle upon Tyne, UK; 3 Guy's & Thomas’ NHS Foundation Trust, London, UK; 4 NHS Blood and Transplant, Bristol, UK

**Keywords:** TRANSPLANT SURGERY

## Abstract

**Introduction:**

Ex vivo normothermic perfusion (EVNP) is a novel technique that reconditions the kidney and restores renal function prior to transplantation. Phase I data from a series of EVNP in extended criteria donor kidneys have established the safety and feasibility of the technique in clinical practice.

**Methods and analysis:**

This is a UK-based phase II multicentre randomised controlled trial to assess the efficacy of EVNP compared with the conventional static cold storage technique in donation after circulatory death (DCD) kidney transplantation. 400 patients receiving a kidney from a DCD donor (categories III and IV, controlled) will be recruited into the study. On arrival at the transplant centre, kidneys will be randomised to receive either EVNP (n=200) or remain in static cold storage (n=200). Kidneys undergoing EVNP will be perfused with an oxygenated packed red cell solution at near body temperature for 60 min prior to transplantation. The primary outcome measure will be determined by rates of delayed graft function (DGF) defined as the need for dialysis in the first week post-transplant. Secondary outcome measures include incidences of primary non-function, the duration of DGF, functional DGF defined as <10% fall in serum creatinine for 3 consecutive days in the first week post-transplant, creatinine reduction ratio days 2 and 5, length of hospital stay, rates of biopsy-proven acute rejection, serum creatinine and estimated glomerular filtration rate at 1, 3, 6 and 12 months post-transplant and patient and allograft survival. The EVNP assessment score will be recorded and the level of fibrosis and inflammation will also be measured using tissue, blood and urine samples. Ethics and dissemination. The study has been approved by the National Health Service (NHS) Health Research Authority Research Ethics Committee. The results are expected to be published in 2020.

**Trial registration number:**

ISRCTN15821205; Pre-results.

**Strengths and limitations of this study**

- This is a large multicentre randomised controlled trial. It will provide evidence of the effect of ex vivo normothermic perfusion (EVNP) on early graft function in donation after circulatory death kidney transplantation.
- The secondary objectives will allow us to examine the impact of EVNP on rates of acute rejection and longer term graft function and survival.
- Tissue, blood and urine samples will enable a more in-depth analysis of the effects of EVNP on ischaemia reperfusion.
- The limitation of this study is the lack of blinding. Owing to the nature of the technique and in order to report safety outcomes, it is not possible to blind the surgical team to the allocation of the kidney.
- Delayed graft function, defined as dialysis within the first week of transplantation, is subjective. However, it is the best accepted measure of early graft function.

## Introduction

Kidney transplantation continues to be limited by a shortage of organ donors. In response to this, there has been an increase in the use of kidneys from marginal donors and a significant proportion of transplant kidneys are now provided by donation after circulatory death (DCD) donors.^1^ DCD kidneys inevitably sustain a warm ischaemic insult prior to retrieval and 50–60% of these kidneys have delayed graft function (DGF) after transplantation.^1^ DGF is associated with an increased risk of acute rejection, longer in-patient stay and therefore greater cost, and may also reduce allograft survival.^1–7^ Moreover, when compared with standard criteria donors, DCD kidneys are three times more likely to be declined for transplantation due to concerns over their quality.^8^ ^9^

Organ preservation has traditionally relied on hypothermic techniques based on the principle that refrigeration reduces cellular metabolism and tissue oxygen requirements. The problem is that in the anoxic hypothermic environment anaerobic cellular respiration continues, albeit at a slow pace. Oxidative phosphorylation is uncoupled and mitochondrial ATP production by chemiosmosis ceases.^10^ ATP continues to be generated at a much slower rate by substrate-based phosphorylation in the glycolytic pathway. This leads to depletion of intracellular ATP stores. Anaerobic respiration generates lactic acid leading to worsening intracellular acidosis and eventually loss of cell viability.^10^ The initial warm ischaemic injury sustained by DCD kidneys makes them less tolerant of cold ischaemic injury during hypothermic preservation.^11^ ^12^

Normothermic perfusion techniques offer an alternative form of organ preservation and resuscitation that has the potential to limit some of the effects of hypothermic preservation techniques.^13^ Ex vivo normothermic perfusion (EVNP) is a novel technique that may help to recondition ischaemically injured kidneys prior to transplantation.^13^ The aim of EVNP is to restore metabolism and function to the kidney prior to transplantation by circulating a warm oxygenated red cell-based solution through the kidney.

EVNP has recently been introduced into clinical practice for kidneys from marginal donors.^13–17^ The early experience of renal transplantation after EVNP shows that the technique is feasible, safe and may improve early graft function.

This study will investigate the effect of 60 min EVNP in kidneys from DCD donors compared with the traditional technique of static cold storage.

The primary outcome measure will be DGF defined as the need for dialysis in the first 7 days post-transplantation. Secondary outcome measures will include more sensitive measures of renal function over the first 7 days post-transplant, length of hospital stay, graft and patient survival at 1 year and rates of acute rejection. Fibrosis, inflammatory markers and urinary biomarkers will also be examined in tissue, blood and urine.

## Methods

### Study type

**Figure 1:**
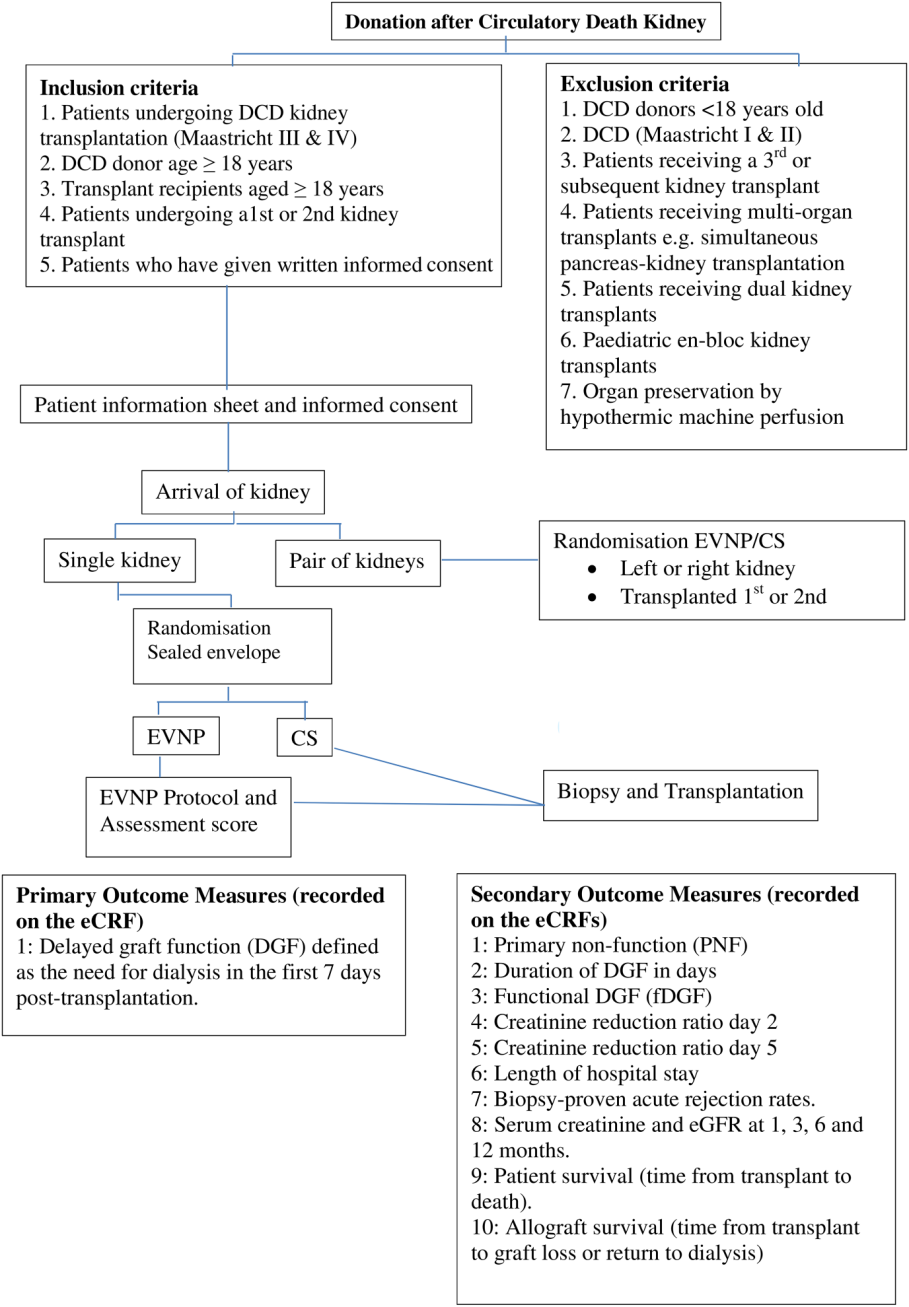
Trial design. CS, cold storage; DCD, donation after circulatory death; DGF, delayed graft function; eCRF, electronic case report form; eGFR, estimated glomerular filtration rate; fDGF, functional DGF; EVNP, ex vivo normothermic perfusion.

A randomised controlled, open-label trial of the effect of EVNP on initial graft function in DCD kidney transplant. Figure 1 summarises the design of the trial.

### Eligibility

Patients will be eligible for the trial if they meet the following criteria: patients undergoing a DCD kidney transplantation (Maastricht categories III and IV); the donor and recipient must be ≥18 years and it must be the patient's first or second kidney transplant.

Written informed consent will be taken from all patients included in the trial by a qualified member of the study team. Patients will not be eligible if the donor or recipient is under the age of 18 years, the DCD donor is in Maastricht categories I and II, patients receiving a third or subsequent kidney transplant, patients receiving multiorgan transplants, for example, simultaneous pancreas–kidney transplantation, patients receiving dual kidney transplants, paediatric en-bloc kidney transplants or kidneys that have been preserved by hypothermic machine perfusion. There will be four participating centres in the UK: Cambridge (lead), Newcastle, Guy's and Edinburgh.

### Randomisation

Patients will be allocated at random in a 1:1 ratio to either static cold storage (CS) plus 1 hour of EVNP (n=200) or CS (n=200) only. A patient information sheet will be given to potential recruits on admission for DCD kidney transplantation and written informed consent will then be obtained (see online supplementary appendices 1 and 2). Randomisation will be performed after the transplant recipient and kidney have both arrived in the transplanting centre and a final decision to proceed with transplantation has been made by a member of the study team. The randomisation will be performed on a web-based system (sealed envelope) which uses a computer-generated randomisation sequence. In cases where paired kidneys from the same donor are transplanted in the same centre, one kidney from the pair will be randomly allocated to CS and the other to EVNP. In these cases, the randomisation will also determine which kidney (right or left) will be transplanted first. Owing to the nature of the trial, no one is blinded to treatment allocation.

10.1136/bmjopen-2016-012237.supp1 supplementary appendix

10.1136/bmjopen-2016-012237.supp2 supplementary appendix

### Multiple vessels

DCD kidneys with multiple renal arteries will not be excluded from the trial as EVNP is technically possible in such kidneys. A cannula that preserves the carrel patch or a graft reconstruction using donor vessels will be used where possible. Nonetheless, EVNP may prove to be very difficult in kidneys with particularly complex vascular anatomy. If such a kidney is randomised to EVNP, then the local investigator may decide to use CS alone. Nonetheless, the recipient will be analysed in the EVNP arm of the study in line with the intention-to-treat basis. As per protocol, analysis will also be performed to assess the effects of the actual preservation method.

### Kidney retrieval

All kidneys will be retrieved by the UK national retrieval teams. Following in situ flushing of the abdominal organs with hyperosmolar citrate (HOC) solution or University of Wisconsin (UW) solution, kidneys will be removed and then placed individually in preservation solution and packed in ice. The perfusion solution used will be recorded.

### vivo normothermic perfusion

The EVNP circuit has been designed using paediatric cardiopulmonary bypass technology (Medtronic, Watford, UK) and consists of a centrifugal blood pump (Bio-pump 560), a heat exchanger, a venous reservoir (Medtronic), 1/4 inch Polyvinyl chloride (PVC) tubing and an Affinity membrane oxygenator (Medtronic). The hardware includes a speed controller, a TX50P flow transducer and a temperature probe (Cole-Parmer, London, UK). Two infusion pumps are also incorporated into the system.

### Perfusate

The circuit will be primed with perfusate solution (Ringer's solution, Baxter Healthcare, Thetford, Norfolk, UK) and one unit of O-positive or O-negative packed red cells from the blood bank. Mannitol 10% (Baxter Healthcare), dexamethasone 8 mg (Organon Laboratories, Cambridge, UK) and heparin (CP Pharmaceuticals, Wrexham, UK) will be added to the perfusate. Sodium bicarbonate 8.4% (Fresenius Kabi, Cheshire, UK) will be added to normalise the pH. A nutrient solution (Synthamin 17, Baxter Healthcare) with sodium bicarbonate 8.4%, insulin (Novo Nordisk, Denmark) and multivitamins (Cernevit, Baxter Healthcare) will be infused into the circuit at a rate of 20 mL/hour. Prostacyclin 0.5 mg (Flolan, Glaxo-Wellcome, Middlesex, UK) will be infused into the arterial arm of the circuit at a rate of 5 mL/hour and glucose 5% (Baxter Healthcare) at 5 mL/hour. Ringer's solution will be used to replace urine output mL for mL.

### Perfusion chamber

Kidneys undergoing EVNP will be placed in a custom-designed sterile perfusion chamber and the renal artery and vein will be cannulated and primed with cold 0.9% sodium chloride.

### Perfusion parameters

Kidneys will be perfused at a set mean arterial pressure (75 mm Hg). The plasma-free red cell-based perfusate will be circulated from the venous reservoir through the centrifugal pump into the membrane oxygenator, where it is oxygenated and also warmed to 35–36^°^C. It will then flow through the arterial limb of the circuit to the renal artery. Venous return from the renal vein will be fed back into the reservoir.

Renal blood flow (RBF) will be monitored continuously during EVNP. Intrarenal resistance will be calculated (mean arterial pressure/RBF) every 5 min until the end of perfusion. The total urine output will be recorded. Blood gas analysis will be used to measure the acid–base balance pre-EVNP and post-EVNP.

### Postperfusion

After EVNP, kidneys will be flushed with ∼500 mL of cold (4^°^C) HOC (Baxter Healthcare, UK) to remove the perfusate and then placed back in ice until transplanted. Prior to transplantation, the arterial Carrel patch may have to be excised along with a short segment of vein in order to remove the cannula ligature sites.

### EVNP assessment scoring

An assessment score will be recorded for all kidneys undergoing EVNP but will not be used to make decisions about the suitability of kidneys for transplantation (table 1).^13^

**Table 1:** Ex vivo normothermic perfusion (EVNP) score

EVNP score	
Macroscopic appearance	Points
Excellent perfusion with global and even pink appearance	1 point
Moderate perfusion with some areas of patchy or mottled perfusion	2 points
Poor perfusion with a globally mottled and purple appearance	3 points
Mean renal blood flow (mL/min/100 g) <50 mL/min/100 g	1 point
Mean renal blood flow (mL/min/100 g) >50 mL/min/100 g	0 point
Total urine output (mL) <43mL/hour	1 point
Total urine output (mL) >43mL/hour	0 point

Scores for macroscopic appearance, renal blood flow and urine output will be added to yield an overall assessment score ranging from 1 (the highest quality) to 5 (the lowest quality).

### Transplantation

This will be performed using standard techniques. Anaesthesia will be given according to local protocols. Kidneys will be transplanted into either iliac fossa with anastomosis of the artery to the common, external or internal iliac arteries. The vein will be anastomosed to either the common or the external iliac vein. The ureteric anastomosis will be performed as an extravesical onlay over a double J stent.

### Immunosuppressive therapy

All centres will use similar immunosuppressive protocols as follows: patients will receive 20 mg of basiliximab intravenously pretransplant and 20 mg intravenously on postoperative day 4. Patients will receive a bolus of methylprednisolone intravenously at induction of anaesthesia using a dosage according to local practice. The post-transplant oral prednisolone regimen will also be according to local practice.

Patients may be treated with either tacrolimus or cyclosporine according to local protocols. There will be no restriction on the formulation of the prescribed calcineurin inhibitors. Tacrolimus will be prescribed to achieve target trough levels of 3–12 ng/mL. Cyclosporine will be prescribed to achieve target trough levels of 100–250 ng/mL. The first dose of calcineurin inhibitor may be given preoperatively or postoperatively. All patients will receive mycophenolate as either mycophenolate mofetil (Cellcept) at a starting dose of at least 1 g/day or mycophenolate sodium (Myfortic) starting at a dose of at least 720 mg/day.

Antimicrobial and antithrombotic prophylaxis will be given according to local protocols. It is expected that patients will also receive prophylaxis against *Pneumocystis jirovecii* pneumonia, oral candidiasis and cytomegalovirus (valganciclovir for 100 days in CMV-positive donor to CMV-negative recipient transplants).

### Outcomes

#### Primary outcome measure

The primary outcome measure is DGF defined as the need for dialysis in the first 7 days post-transplantation.^18^

#### Secondary outcome measures

The secondary outcome measures include incidences of primary non-function (PNF) defined as the permanent lack of allograft function from the time of transplantation (This will include graft losses due to irreversible rejection and vascular thrombosis. The cause of graft loss will be recorded.); the duration of DGF in days; functional DGF (fDGF) defined as <10% fall in serum creatinine for 3 consecutive days in the first week post-transplant; creatinine reduction ratio day 2 (CRR2=creatinine day 1−creatinine day 2/creatinine day 1) and CRR5 (CRR5=pretransplant creatinine−creatinine day 5/pretransplant creatinine);^19^ length of hospital stay, rates of biopsy-proven acute rejection rate and measures of renal function (serum creatinine and estimated glomerular filtration rate (eGFR)) at 1, 3, 6 and 12 months post-transplant. Patient survival (time from transplant to death) and allograft survival (time from transplant to graft loss or return to dialysis) will be recorded. The EVNP score will be calculated for each kidney undergoing EVNP (table 1). The predictive value of this assessment score on graft function and outcome will be evaluated. A clinical decision on the suitability of the kidney for transplantation will be made using normal criteria prior to perfusion. However, the EVNP assessment score will be taken into consideration. Arterial and venous samples will be collected during perfusion and used to measure oxygen consumption, acid–base balance and concentrations of sodium, potassium, glucose, lactate and calcium. Concentrations of sodium and potassium will also be measured in the urine at the end of EVNP.

### Renal fibrosis

One of the most common causes of graft failure after transplantation is the development of chronic allograft nephropathy.^20^ The aim of this study is to determine if EVNP can slow the progression of fibrosis. Biopsies of the kidney will be taken pretransplant and after 3 months. Biopsies will be fixed in formalin and paraffin embedded cut sections of the graft will be stained with sirius red (stains for collagen III). The degree of fibrosis will be quantified using computerised digital image analysis.

### Injury markers

Ischaemia reperfusion injury is a leading cause of early graft dysfunction. The aim of this study is to determine if EVNP can reduce the amount of inflammation and injury after transplantation. Blood and urine samples will be collected pretransplant and post-transplant and used to measure inflammation (inflammatory cytokines: interleukin-6 (IL-6), tumour necrosis factor-α, IL-8) and kidney injury (neutrophil gelatinase-associated lipocalin, liver fatty acid binding protein). Further biopsies of the kidney will be taken and snap frozen in liquid nitrogen or stored in RNA later, preimplantation and 30 min post-transplant. These will be used for light microscopy and RNA sequencing to determine the degree of acute tubular injury, measure inflammatory cytokines and downstream signalling molecules such as vascular endothelin growth factor (VEGF) and Erythropoietin (EPO).

### Sample storage

Samples will be stored in the clinical and research laboratories within the participating centres. Samples will be coded and donor identifiable material will only be available to the principal investigator (PI) and the research team at the participating centres. Samples are appropriately labelled in accordance with the trial procedures to comply with the 1998 Data Protection Act. Biological samples collected from participants as part of this trial will be transported, stored, accessed and processed in accordance with national legislation relating to the use and storage of human tissue for research purposes and such activities shall at least meet the requirements as set out in the 2004 Human Tissue Act and the 2006 Human Tissue (Scotland) Act.

On completion of the trial, samples will be disposed of in accordance with the Human Tissue Authority's Code of Practice.

### Statistics

#### Sample size determination

The trial size was calculated with respect to the primary end point, which is DGF defined as the requirement for dialysis in the first 7 days post-transplantation. Based on 5 years of data from the three participating centres, the overall rate of DGF in DCD kidney transplants is 50%. In a pilot series of kidney transplants from extended criteria donors (ECD), 18 kidneys undergoing CS followed by 1 hour of EVNP were compared with a historical control group of 47 ECD transplants after CS alone. The DGF rates were 1/18 (6%) in the EVNP group compared with 17/47 (36%) in the CS group.^14^

Using a fixed sample size study, with interim analyses after 125 and 250 patients have been enrolled and reached 365 days post-transplant, a total of 376 patients receiving a DCD kidney (Maastricht categories III and IV controlled donors) will be required to detect a 30% relative reduction in DGF (from 50% to 35%) with a power of 80% and a statistical significance of α=0.05. To allow for a study withdrawal rate of 6%, a total of 400 patients will be recruited.

#### Interim analysis

Two interim analyses will be performed during the study period. The first of these will be after 125 patients have received a transplant and reached 7 days post-transplant and the second after 250 patients have been recruited (and have received a transplant and reached 7 days post-transplant).

#### Analysis population and principles

The population used for efficacy analyses will be an intent-to-treat population including all eligible randomised patients. This will be the primary analysis for the trial. Characteristics of all randomised patients will be tabulated by arm of the trial to describe the cohort.

The primary and secondary outcomes will also be analysed per protocol.

### Analysis of primary and secondary outcomes

The analyses will be described in detail in a full statistical analysis plan. This section summarises the main issues. A full statistical analysis plan will be drawn up prior to the first interim analysis.

#### Primary analysis

The number of patients who experience DGF between the two groups will be compared using a logistic regression model adjusting for cold ischaemic time and donor age.

#### Secondary outcomes

*PNF*: The number of patients experiencing PNF in each arm will be compared using Fisher's exact text. The cause of graft loss will be tabulated by arm. The duration of DGF in days will be summarised using the Kaplan-Meier estimate of the duration of DGF for all those who experienced DGF and compared using a log-rank test.

*Outcome measures*: The number of patients that experience functional DGF will be compared using Fisher's exact test. The CRR2 and CRR5 will be compared by arm using a two-sample t-test. The length of hospital stay, defined from admission to discharge, will be estimated using the Kaplan-Meier method, with deaths in hospital censored, and compared between arms using the log-rank test.

*Biopsy-proven acute rejection rate*: The number of patients in each arm will be compared using Fisher's exact test. Longitudinal changes in serum creatinine and eGFR will be separately assessed at 1, 3, 6 and 12 months post-transplant and mean values compared using two-way analysis of variance with repeated measures for patients in each arm of the trial. Any difference between arms in the way serum creatinine and eGFR change over time will also be assessed using this model. Patient survival (time from transplant to death) and allograft survival (time from transplant to graft loss or return to dialysis) will be analysed using a Kaplan-Meier estimate of the probability of an event after 12 months and a log-rank test. Time to graft loss or return to dialysis will be modelled.

### Analysis population and missing data

Normally if a patient experiences DGF, they would remain in hospital for a minimum of 7 days. Hence, for pragmatic reasons, if a patient is discharged from the hospital prior to 7 days post-transplant and the patient is known to be alive at 7 days post-transplant, then such patients will be imputed to have not experienced DGF. Some patients may also withdraw consent, be withdrawn or die during the conduct of the trial. In any of these cases, it is impossible to know whether such patients are either less or more likely to experience DGF had they not experienced early censoring. Sensitivity analyses will be performed to account for this.

### Safety outcomes

Incidences of acute rejection, renal artery or renal vein thrombosis, complications of the renal transplant biopsy and the number of hospital admissions for any recognised complication of renal transplantation and immunosuppression will be summarised by arm.

The University of Cambridge and Cambridge University Hospital National Health Service (NHS) Foundation Trust will be the sponsor for this study. A Trial Steering Committee will meet six monthly to review the trial, monitor recruitment rates to consider protocol amendments and provide advice. A Trial Management Group will be responsible for the Ethics Committee application and the production of reports and the day-to-day running of the trial. A Data Monitoring Committee will consist of three independent members with expertise in renal transplantation, clinical trials and statistics. They will meet at least annually to review data, primarily concentrating on patient safety.

### Data handling

A data will be collected prospectively and recorded on the electronic case report forms. The data will be monitored by the trial manager and audited each month. Each participant will be allocated a unique study number and will be identifiable by this number throughout the course of the study. This number will be used on all documentation and during analysis of the results. Tissue, blood and urine samples will be coded with a unique number. Any data transferred will be carried out under the NHS Code of Practice on Confidentiality.

### Ethical considerations

The study protocol and trial documents including the consent form and participant information sheet have been approved by the NHS Health Research Authority East of England, Cambridge Central Research Committee (15/EE/0356). Approval has also been granted by the NHS Research & Development (R&D) department and will be sought at each participating centre. Substantial amendments will require ethical review. Amendments will also need to be reviewed and accepted by the NHS R&D departments before they can be implemented in practice at sites.

Insurance of negligent or non-negligent harm will be covered under the University’s clinical trial policy.

There are no conflicts of interest for the PIs or members of the study team.

### Dissemination

The results of the study will be submitted for peer review for publication in a scientific journal and will also be presented at national and international meetings.

## References

[1] Summers DM, Watson CJ, Pettigrew GJ Kidney donation after circulatory death (DCD): state of the art. Kidney Int 2015;88:241–9. 10.1038/ki.2015.88 25786101

[2] Saidi RF, Elias N, Kawai T Outcome of kidney transplantation using expanded criteria donors and donation after cardiac death kidneys: realities and costs. Am J Transplant 2007;7:2769–74. 10.1111/j.1600-6143.2007.01993.x 17927805

[3] van der Vliet JA, Warle MC, Cheung CL Influence of prolonged cold ischemia in renal transplantation. Clin Transplant 2011;25:E612–16. 10.1111/j.1399-0012.2011.01510.x 21919965

[4] Snoeijs MG, Winkens B, Heemskerk MB Kidney transplantation from donors after cardiac death: a 25-year experience. Transplantation 2010;90:1106–12. 10.1097/TP.0b013e3181f83b0b 20861804

[5] Yarlagadda SG, Coca SG, Formica RN Jr, et al Association between delayed graft function and allograft and patient survival: a systematic review and meta-analysis. Nephrol Dial Transplant 2009;24:1039–47. 10.1093/ndt/gfn667 19103734

[6] Rao PS, Ojo A The alphabet soup of kidney transplantation: SCD, DCD, ECD—fundamentals for the practicing nephrologist. Clin J Am Soc Nephrol 2009;4:1827–31. 10.2215/CJN.02270409 19808229

[7] Quiroga I, McShane P, Koo DD Major effects of delayed graft function and cold ischaemia time on renal allograft survival. Nephrol Dial Transplant 2006;21:1689–96. 10.1093/ndt/gfl042 16490743

[8] Dare AJ, Pettigrew GJ, Saeb-Parsy K Preoperative assessment of the deceased-donor kidney: from macroscopic appearance to molecular biomarkers. Transplantation 2014;97:797–807. 10.1097/01.TP.0000441361.34103.53 24553618

[9] Callaghan CJ, Harper SJ, Saeb-Parsy K The discard of deceased donor kidneys in the UK. Clin Transplant 2014;28:345–53. 10.1111/ctr.12319 24506794

[10] Nourbakhsh N, Singh P Role of renal oxygenation and mitochondrial function in the pathophysiology of acute kidney injury. Nephron Clin Pract 2014;127:149–52. 10.1159/000363545 25343840 PMC5540439

[11] Summers DM, Johnson RJ, Allen J Analysis of factors that affect outcome after transplantation of kidneys donated after cardiac death in the UK: a cohort study. Lancet 2010;376:1303–11. 10.1016/S0140-6736(10)60827-6 20727576

[12] Summers DM, Johnson RJ, Hudson A Effect of donor age and cold storage time on outcome in recipients of kidneys donated after circulatory death in the UK: a cohort study. Lancet 2013;381:727–34. 10.1016/S0140-6736(12)61685-7 23261146

[13] Hosgood SA, Nicholson ML First in man renal transplantation after ex vivo normothermic perfusion. Transplantation 2011;92:735–8. 10.1097/TP.0b013e31822d4e04 21841540

[14] Nicholson ML, Hosgood SA Renal transplantation after ex vivo normothermic perfusion: the first clinical study. Am J Transplant 2013;13:1246–52. 10.1111/ajt.12179 23433047

[15] Hosgood SA, Nicholson ML The first clinical case of intermediate ex vivo normothermic perfusion in renal transplantation. Am J Transplant 2014;14:1690–2. 10.1111/ajt.12766 24816186

[16] Hosgood SA, Nicholson ML Ex vivo normothermic perfusion of declined human kidneys after inadequate in situ perfusion. Am J Transplant 2014;14:490–1. 10.1111/ajt.12568 24330455

[17] Hosgood SA, Barlow AD, Hunter JP Ex-vivo normothermic perfusion—an innovative technology for quality assessment of marginal donor kidney transplants. Br J Surg 2015;102:1433–40. 10.1002/bjs.9894 26313559

[18] Mallon DH, Summers DM, Bradley JA Defining delayed graft function after renal transplantation: simplest is best. Transplantation 2013;96:885–9. 10.1097/TP.0b013e3182a19348 24056620

[19] Vilar E, Varagunam M, Yaqoob MM Creatinine reduction ratio: a useful marker to identify medium and high-risk renal transplants. Transplantation 2010;89:97–103. 10.1097/TP.0b013e3181be3dd1 20061925

[20] Boor P, Floege J Renal allograft fibrosis: biology and therapeutic targets. Am J Transplant 2015;15:863–86. 10.1111/ajt.13180 25691290

