# Supplementary material for: Protocol of a randomised controlled, open-label trial of ex vivo normothermic perfusion versus static cold storage in donation after circulatory death renal transplantation
Source: BMJ Open. 2017 Jan 23;7(1):e012237. doi: 10.1136/bmjopen-2016-012237 (PMC5278243; doi:10.1136/bmjopen-2016-012237)
Supplement: supplementary appendix [file bmjopen-2016-012237supp_appendix1.pdf]

Pre-Transplant Patient Information Sheet

You are being invited to take part in a research study. Before you decide, it is important for you to understand why the research is being done and what it will involve. Please take time to read the following information carefully and discuss it with others if you wish. Ask us if there is anything that is not clear or if you would like more information. Take time to decide whether or not you wish to take part.

Study Title: Ex-vivo Normothermic Perfusion Trial

Summary
We would like to assess whether a new technique of kidney preservation can improve early graft function compared to the standard cold storage technique.

1 What is the purpose of the study?

When kidneys are removed from an organ donor they are normally stored on ice until they are ready to be transplanted. A kidney can be preserved safely at a low temperature in these conditions. However, there is some degree of deterioration and the longer they are left in this condition the more they deteriorate (rather like food that is kept in the fridge). We have developed a technique that may improve the quality of the kidney. This involves placing the kidney on a machine and passing a warmed, oxygen-rich solution containing red blood cells through it for about one hour. Under these conditions the kidney can start to function again and produce urine. This improves the condition of the kidney and may improve function after transplantation.

2 How is this study designed?

This study will assess the technique of warming a kidney and compare it to the standard cold storage technique that is commonly used. Kidneys will be randomly assigned to receive either the warming technique or standard cold storage.

3 Why have I been invited?

The kidney you are being offered is a suitable match for you. It is a kidney from a donation after circulatory death donor and meets the criteria for entry into the trial.

4 Do I have to take part?

It is completely your choice whether you want to take part. If you decline to be included in the study the kidney will undergo the standard cold storage technique of preservation with no intervention.

5 What will happen to me if I take part?

You will be prepared for surgery in the normal way. Standard practice involves keeping the transplant kidney under cold storage in ice until the time of the transplant operation. If you consent to take part in this trial then your kidney will be randomly assigned to either standard practice or to having warm perfusion for one hour immediately before the kidney is transplanted. Should there be any problem with the warm perfusion procedure then the kidney can be quickly removed from the perfusion machine and returned to cold storage in

ice before transplantation. This failsafe position has not been required so far in our first 40 cases.
During the transplant operation we will also take a small tissue biopsy from the kidney 30 minutes after it has been transplanted. Although there is a small risk of causing bleeding from the kidney biopsy site (<5%) your surgeon will be able to repair the bleeding site if this happens.

6 What do I have to do?
After your transplant you will receive the normal standard care but will also be asked to provide a few additional blood and urine samples for analysis. Your participation in this study will not affect the way you are followed up after a transplant. The normal follow up involves clinical visits at least twice a week for six weeks and then weekly for a further six weeks.
At three months after your transplant we will plan to perform a routine needle biopsy of the kidney to look for scarring. This would help us with our research but it is not compulsory. Needle biopsies have a good safety record but there is a small risk (less than 1%) of significant bleeding.
After three months you will be seen either in Cambridge or your local centre. The length between clinical visits will be gradually extended after the first three months.

7 What are the alternatives for treatment?
If you decide not to take part in the trial the kidney will not receive any intervention and will be transplanted as normal.

8 What are the possible disadvantages and risks of taking part?
There are no potential side effects to you. This technique of perfusion is applied to the kidney only, before it is transplanted. There is a small risk that the kidney might be damaged during the assessment and therefore could not be transplanted. This has not happened in our experience of 40 cases so far but it remains as a potential risk. Although your kidney may be deemed suitable for transplantation it is a kidney from a marginal donor and we know that this increases the rate of delayed graft function. This means that it may take some time for the kidney to start to function fully. There is also a small risk that the kidney will never function.

9 What are the possible benefits of taking part?
This trial is being performed because we are uncertain whether or not warm perfusion improves the outcome of kidney transplantation. Our experience so far suggests that using this technique to recondition the kidney can result in better postoperative function. It is also possible however that warm perfusion worsens the outcome of a kidney transplant.

10 What happens when the research study stops?
At the end of the research study you will continue to be followed up for your kidney transplant either at Addenbrooke's Hospital or at your local renal hospital.

10 What if new information becomes available?
If new information becomes available your Transplant Consultant might consider it to be in your best interests to withdraw you from the study. He/she will explain the reasons and arrange for your care to continue.

12 What will happen if I do not want to carry on with the study?
You will be given normal care after the kidney transplant. If you withdraw from the study we will ask permission to use the data collected up to your withdrawal.

13 What will happen to the samples I give?
All urine, blood and tissue samples requested following renal transplantation, either routinely or because of a clinical need, will be tested or disposed of according to normal hospital policies and procedures. We will keep a small amount of these samples within the Hospital in a secured area for analysis at a later date. Once analysed, these will be disposed of according to normal hospital policy. Tissue samples will also be kept within the hospitals pathology department.

14 What will happen to the results of the research study?
The results of the research will be published in specialist journals in order to inform other transplant doctors around the world. You will not be identified in any report or publication. You will be able to get a copy of the results by asking the kidney doctors in the follow up clinic.

15 Will my General Practitioner/Family Doctor (GP) be involved?
Participation in this trial will not affect your treatment and follow-up by your GP after discharge from the hospital.

16 Who is organising the research?
The research is being organised by doctors at the Cambridge Transplant Unit. The trial is funded by Kidney Research UK and approximately 400 patients will be recruited.

17 Who has reviewed the study?
The study has been reviewed by the transplant doctors in Cambridge, Kidney Research UK and the Local Research Ethics Committee.

18 Will my taking part in this study be kept confidential?
We will follow ethical and legal best practice and all information about you will be handled confidentially. If you join the study, some parts of your medical records and the data collected for the study will be looked at by authorised persons from the research team. They may also be looked at by representatives of the regulatory authority or by those responsible for research and development audit (for monitoring the quality of the research). All have a duty of confidentiality to you as a research participant and will do their best to meet this duty. Our procedure for handling, processing, storage and destruction of data will match the *Data Protection Act 1998*. Your name will not be disclosed outside the hospital. The data collected will be stored and retained securely for 10 years and it will also be disposed of

securely. You have the right to check the accuracy of data held about you and correct any errors.

19 What if there is a problem?
Any complaint about the way you have been dealt with during the trial or any possible harm you might suffer will be addressed. If you have any concerns about any aspect of this trial you should speak to your trial doctor who will do their best to answer your questions.

In the event that something does go wrong and you are harmed by taking part in the research and this is due to someone's negligence then you may have grounds for a legal action for compensation against Cambridge University Hospitals NHS Foundation Trust or the University of Cambridge. The normal National Health Service complaints mechanisms will still be available to you (if appropriate). The University has obtained insurance, which provides no-fault compensation i.e. for non-negligent harm, you may be entitled to make a claim for this.

Obtaining further information
If you have any questions or concerns about any aspect of this study, you should ask to speak to the researchers who will do their best to answer your questions or to Professor Michael Nicholson (01223 339221).

Complaints and Independent Advice
If you wish to speak to an independent body about any concerns or complaints about any aspect of the way you have been approached or treated during this trial, you can do this through the Addenbrooke's Kidney Patient's Association or the Patient Advice and Liaison Service (PALS) at Addenbrooke's Hospital. The formal NHS complaints procedure is also available to you. Details can be obtained through the hospital.

Complaints
If you remain unhappy and wish to complain formally, you can do this through the NHS complaints procedure.

NHS based research
If you are harmed by taking part in this research project, there are no special compensation arrangements. If you are harmed due to someone's negligence, then you may have grounds for a legal action but you may have to pay for it. Regardless of this, if you wish to complain, or have any concerns about any aspect of the way you have been approached or treated during the course of this study, the normal National Health Service complaints mechanisms will be available to you.

Contacts for further information
A) General information about research can be found on www.nres.org.uk; www.addenbrookes.nhs.uk; or www.instituteofclinicalresearch.org.uk

B) For specific information about this research project, contact Professor Michael Nicholson, 01223 339221.

C) For advice on whether you should participate in the study, contact either Professor Michael Nicholson on 01223 339221, or one of the other transplant doctors on G5 on 01223 217306.

D) For independent advice you can also contact the Patient Advice and Liaison Service (PALS) on 01223 216 756, email pals@addenbrookes.nhs.uk

E) Addenbrooke's Kidney Patient's Association email info@akpa.org.uk

Thank you for reading this information sheet and for considering taking part in this study. If you agree to take part you will be given a copy of this information sheet and a signed copy of your consent form to keep.
