# Supplementary material for: Protocol of a randomised controlled, open-label trial of ex vivo normothermic perfusion versus static cold storage in donation after circulatory death renal transplantation
Source: BMJ Open. 2017 Jan 23;7(1):e012237. doi: 10.1136/bmjopen-2016-012237 (PMC5278243; doi:10.1136/bmjopen-2016-012237)
Supplement: supplementary appendix [file bmjopen-2016-012237supp_appendix2.pdf]

Patient Consent Form

Patient Identification Number:

Ex-vivo Normothermic Perfusion Kidney Trial

1. I confirm that I have read and understood the information sheet for the above study. I have had the opportunity to consider the information, ask questions and have had these answered satisfactorily.

   Initial ☐

2. I understand that my participation is voluntary and that I am free to withdraw at any time without giving any reason, without my medical care or legal rights being affected.

   Initial ☐

3. I understand that relevant sections of my medical notes and data collected during the study, may be looked at by individuals from the Cambridge University Hospitals NHS Foundation Trust, where it is relevant to my taking part in this research. I give permission for these individuals to have access to my medical records.

   Initial ☐

4. I agree for the storage and analysis of my serum, urine and kidney biopsy samples. I understand that these samples may be kept for analysis at a later date.

   Initial ☐

5. I agree to take part in the above study.

   Initial ☐

Name of Participant ………………………………………………………………………………………….

Signature…………………………………………………………………… Date……………………………

Name of Person Taking Consent ……………………………………………………………………………

Signature…………………………………………………………………… Date……………………………

Name of Principal Investigator: Professor Michael Nicholson (Consultant Transplant Surgeon)
